# Indirect organogenesis for high frequency shoot regeneration of two cultivars of *Sansevieria trifasciata* Prain differing in fiber production

**DOI:** 10.1038/s41598-022-12640-4

**Published:** 2022-05-20

**Authors:** Eleazar García-Hernández, Maribel M. Loera-Quezada, Dalia C. Morán-Velázquez, Mercedes G. López, Manuel A. Chable-Vega, Alberto Santillán-Fernández, Hilda A. Zavaleta-Mancera, John Z. Tang, Parastoo Azadi, Enrique Ibarra-Laclette, Fulgencio Alatorre-Cobos

**Affiliations:** 1 Colegio de Postgraduados Campus Campeche, Carretera Haltunchén-Edzná km 17.5, Sihochac, 24450 Campeche, México; 2 Centro de Investigación y de Estudios Avanzados del I. P. N., Unidad Irapuato, km 9.6 Libramiento Norte Carretera Irapuato-León, Irapuato, 36821 México; 3 Conacyt-Colegio de Postgraduados Campus Campeche, Carretera Haltunchén-Edzná km 17.5, Sihochac, 24450 Campeche, México; 4 Colegio de Postgraduados, Programa de Posgrado en Botánica y Unidad de Microscopía Electrónica, km 36.5 Carretera México-Texcoco, Montecillo, 56230 México; 5 Complex Carbohydrate Research Center, University of Georgia, Athens, GA 30602 USA; 6 Red de Estudios Moleculares Avanzados, Instituto de Ecología A.C., Xalapa, 91073 México

**Keywords:** Biotechnology, Plant sciences

## Abstract

*Sansevieria trifasciata* is used as an indoor plant, in traditional medicine and as a fiber source. Here we characterized fibers of two of varieties of *S. trifasciata*, Lorentii and Hahnii, and report a protocol for their propagation based on indirect shoot organogenesis. Structural and ribbon fibers were scattered within leaf parenchyma when viewed with confocal laser scanning microscopy. Chemical analysis of the fibers by mass spectrometry and high-performance chromatography revealed higher contents of cellulose and xylose in Lorentii than in Hahnii and significant differences for total lignin between both. A protocol for de novo shoot production was then developed using leaf explants. Time-course histological analyses showed that the first events of transdifferentiation were triggered preferentially in cells surrounding fibers and vascular bundles. Callogenesis and shoot performances were quantified for both varieties, and 2,4-D at 2 and 3 mg·L^-1^ yielded the best results for primary calli induction and fresh calli mass. The length, number, and mass of shoots produced did not differ significantly between the two cultivars. The fast morphogenic response of *S. trifasciata* to in vitro culture may be useful for mass propagation or other biotechnological purposes such as metabolite production.

## Introduction

*Sansevieria* plants are highly popular indoor and outdoor ornamental, succulent plants worldwide. The *Sansevieria* genus (Asparagaceae family) comprises 70 species of herbaceous, perennial plants that originated from Africa, India and Southeast Asia^1,2^. *Sansevieria trifasciata* Prain is the most widely distributed of the species worldwide and presently used to produce commercial cultivars^3,4^. Also known as snake plant, mother in-law’s tongue and viper’s bowstring hemp, *S. trifasciata* has thin, erect, sword-shaped leaves with color combinations that vary by cultivar, but typically the plants are deep green with light gray or yellow stripes^5^.

Snake plants are also cultivated for their medicinal properties. They are used to treat pain and ear inflammation, swelling, bumps, and bruises, infections in boils and in the respiratory tract, colds, diarrhea, coughs, ulcers, and poisonous snake bites^6,7^. Ethnopharmacological studies have confirmed antiulcer, antioxidant, anti-inflammatory, antibacterial, cytotoxic and analgesic properties of ethanolic extracts of *S. trifasciata*^7–9^, which seem to be related to the bioactivity of the flavonoids, steroidal saponins, tannins and cardiac glycosides in leaves, rhizomes and roots^5,6,10,11^.

Snake plants also have a high capacity to reduce environmental pollution produced by gases and heavy metals. For example, two *S. trifasciata* plants in an office can reduce CO_2_ concentrations up to 19%^12^. Volatile organic compounds such as formaldehyde, acetone, benzene, and xylene are also efficiently removed from indoor air by snake plants^13,14^. In recent tests of *S. trifasciata* for removing and bioaccumulating lead (Pb) and cadmium (Cd), high Cd bioconcentration factors were found in roots, and the levels of Pb bioabsorption and accumulation suggest that these plants have a remarkable potential as phytostabilizers in soils contaminated with heavy metals^15–17^.

*Sansevieria* species have traditionally been used as fiber sources in many countries, especially in Africa, but now they are being widely considered as an alternative crop due to their high plasticity for growing in a wide range of adverse conditions (high sunlight, low water availability, poor soils)^2,4,18,19^. Fibers from *S. trifasciata* have intermediate values for density and diameter and good mechanical and thermal properties compared to other herbaceous leaf fibers. Snake plants thus have a high potential for textile and nontextile uses, but more information on their fiber composition is needed to develop industrial applications^2,4,20,21^.

**Figure 1:**
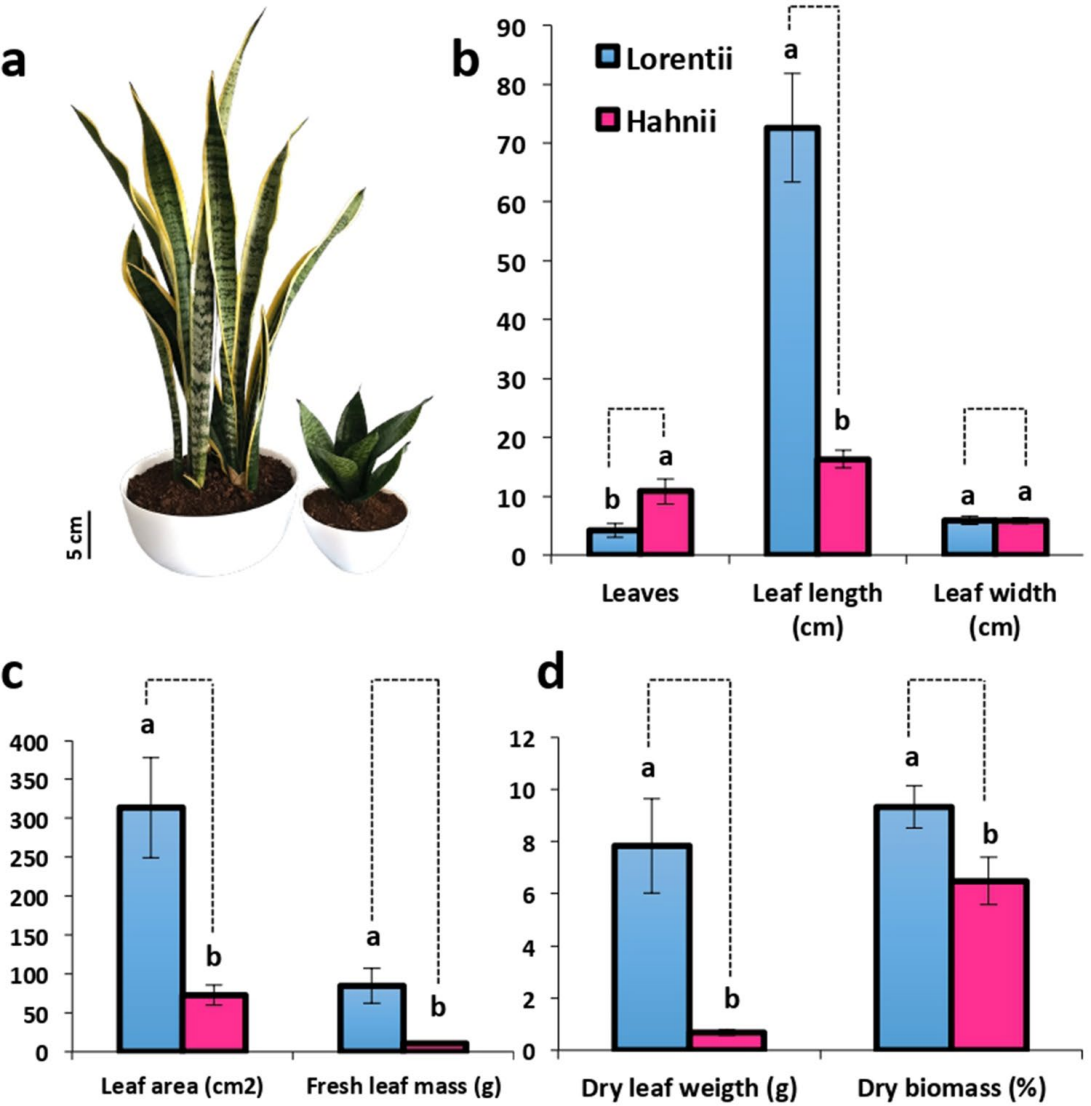
Leaf traits of the two cultivars of *Sansevieria trifasciata*. (**a**) Phenotype of cvs Lorentii (left) and Hahnii (right). Bar = 1 cm. (**b**) Number of leaves/plant, leaf length and width. (**c**) Leaf area and fresh leaf mass. (**d**) Fresh leaf mass and dry biomass. Values are means ± SD. Means with different letters are significantly different according to Tukey’s test (*p* ≤ 0.05).

Snake plants can be easily multiplied by rhizomes or leaf cuttings, but sexual propagation is limited by their rare flowering and inviable seeds^3,22^; thus, genetic improvement by conventional breeding is limited. To overcome this issue and meet the growing commercial demand, mass propagation by in vitro techniques has been explored. But so far only direct and indirect organogenesis have been reported for *S. trifasciata*^22^. Auxin 2,4-D has been effective for inducing callus and meristemoids^23–25^, while 6-benzylaminopurine (BAP) induces high proliferation of shoots^24,25^. Interestingly, indole-3-butyric acid (IBA) combined with a temperature shift during subculturing has been reported as helpful for shoot induction in snake plants^22^. In the present study, we investigated two genotypes of *S. trifasciata* that differ phenotypically (Fig. 1) and in fiber production; cultivars Lorentii and Hahnii. Lorentii is the most common *Sansevieria* cultivar worldwide, while Hahnii is a dwarf Lorentii-derived cultivar^3^ that could be easy to manage and cultivate for laboratory research. First, we analyzed the leaf morphology of adult plants and the chemical composition of their fibers. We then developed a protocol for indirect in vitro organogenesis and histologically described the transdifferentiation events of the shoots produced de novo.

## Results and discussion

### Contrasting leaf morphology and fiber traits of cvs Lorentii and Hahnii

Because *Sansevieria* species have an acaulescent-type growth, here plant height was determined by measuring the length of a fully developed adult leaf. Lorentii plants are 4.6 times taller than Hahnii plants; however, they have fewer leaves (Fig. 1a,b). Consistent with a leaf-dependent plant size, leaf area and fresh/dry mass were significantly greater in Lorentii than in Hahnii (Fig. 1c,d). The results of our morphological characterization of Hahnii are similar to those of tropical dwarf accessions of *Sansevieria* with sizes that range from 8.59 to 12.08 cm^26^. A small size is a key advantage for a model plant species^27,28^. An emerging model for Crassulacean acid metabolism (CAM) plants is *Kalanchoë daigremontiana*^29^, a species with a height up 1 m. *Sansevieria* species, especially its dwarf accessions, may be considered as an alternative option for the study of CAM.

**Figure 2:**
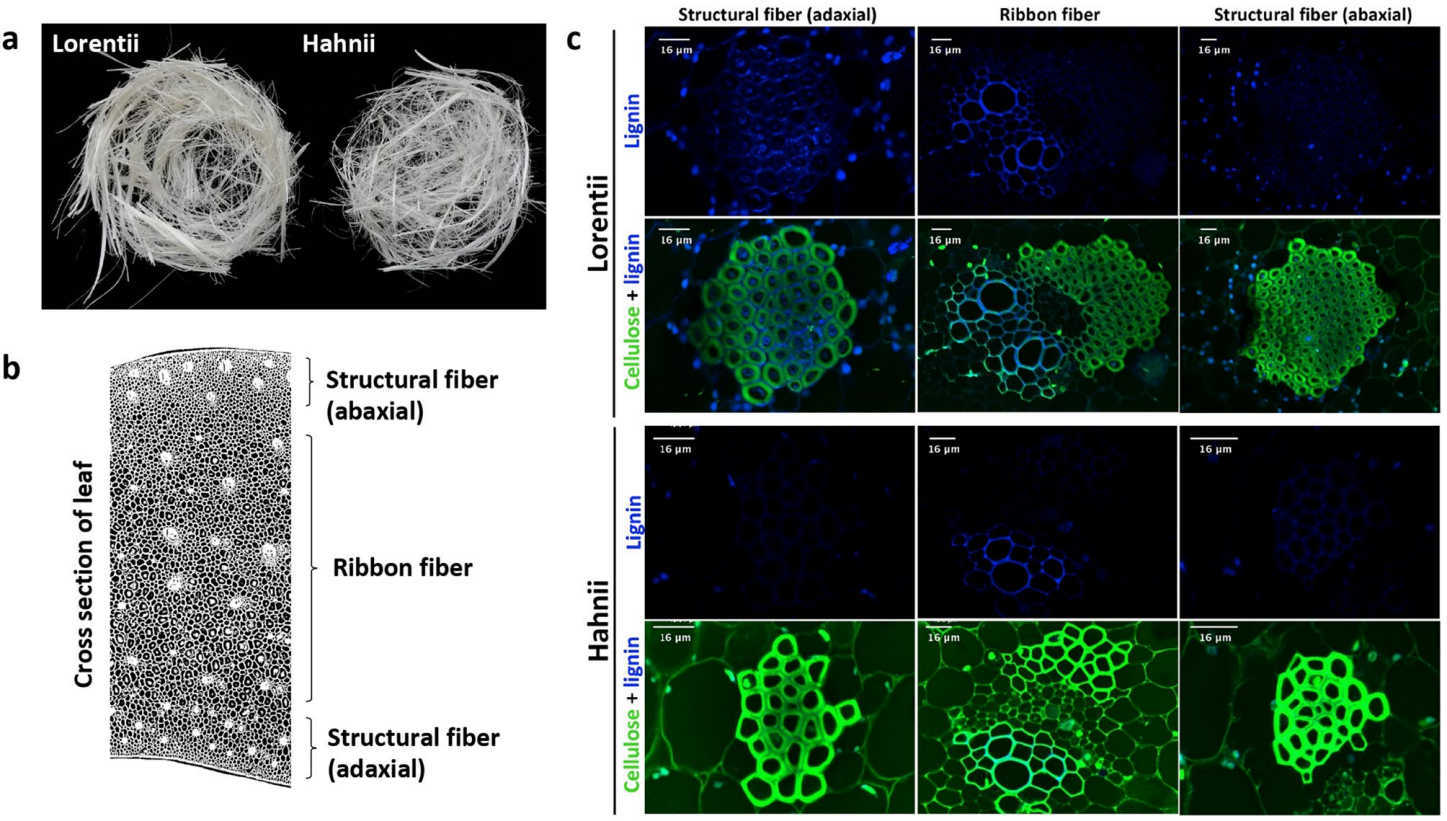
Fibers of the two cultivars of *Sansevieria trifasciata* and images of lignin and cellulose in fibers. (**a**) Crude fibers from cvs Lorentii (left) and Hahnii (right) collected from 3 or 4 adult leaves. (**b**) Micrograph-based drawing of cells in cross section of *S. trifasciata* adult leaf showing the distribution of the different fiber types. (**c**) Confocal laser scanning micrographs of fluorescence of lignin (autofluorescence) and cellulose (stained with propidium iodide) in the different fiber types.

We then examined the morphology of the fibers in leaf cross sections from the two cultivars (Fig. 2a). Two types of fibers were found in both cultivars. Structural fibers were composed of sclerenchyma cells with thickened secondary cell walls that formed polyhedral fiber bundles distributed in peripheral rows in the abaxial and adaxial sides of leaves (Fig. 2b,c). In Lorentii, structural fibers were found as bundles of up to 150 cells. In contrast, in Hahnii, these types of fiber bundles had fewer cells, especially in the abaxial leaf side (≤ 29 cells) (Fig. 2c). Ribbon or arc fibers, scattered in the middle part of the ground tissue of the leaves of both cultivars, had a two-dimensional vascularization pattern as described for succulents^30^ (Fig. 2b). Ribbon fibers form a cap surrounding the vascular bundles; in Lorentii, these caps have up to 93 cells and up to 25 cells in Hahnii. In both cases, ribbon fibers are perivascular sclerenchyma fibers that protect the phloem but are never close to the vascular bundle (Fig. 2c). This fiber morphology for *S. trifasciata* is consistent with that reported for *S. cylindrica*^31^. The variations found in the morphology of structural fibers in the two cultivars of *Sansevieria* analyzed here may be associated with differential development of the parenchyma in unifacial or bifacial leaves, which has been previously observed for Asparagales species, including *Sansevieria*^32,33^. More studies on the biomechanics and functional morphology will be required to know whether and how unifacial (*S. cylindrica*) or bifacial development (*S. trifasciata*) affects the distribution of structural fibers that are thought to provide stiffness to leaf blades in monocots.

Imaging of cellulose and lignin in *Sansevieria* fibers by laser confocal scanning microscopy showed differences between both cultivars analyzed (Fig. 2c). Thus, we quantified the levels and composition of cell wall components in the fibers of Lorentii and Hahnii (Table 1). Cellulose, a homopolymer of glucose monomers, was the major constituent in *Sansevieria* fibers, encompassing up to 41.2% of the dry mass in Lorentii fibers. Lorentii fibers had 1.25 times more cellulose than did Hahnii fibers. For the hemicellulose fraction, xylose was the most abundant monosaccharide in both cultivars, accounting for 11.2% and 8.7% of the dry mass in Lorentii and Hahnii fibers, respectively. Significant amounts of arabinose (up 0.34%) and mannose (0.33%) were also found, especially in cv Hahnii (Table 1). In the evaluation of lignin composition in the raw fibers, the lignin autofluorescence analysis suggested significant differences in lignin content between the *Sansevieria* accessions, which were confirmed by pyrolysis molecular beam mass spectrometry (py-MBMS) (Table 1). The fibers in both cultivars were rich in S-lignin, with contents of the carbohydrate fraction (C5 including xylan and other hemicellulose sugar, and C6 like glucan and cellulose) and total lignin estimated at 1% and 14% of dry mass, respectively (Table 1). Cellulose level obtained here (41.2%) for the two cultivars of *S. trifasciata* was lower than previously reported for *S. trifasciata* (56%), *S. cylindrica* (79.7%), *S. ehrenbergii* (80%) and *S. roxburghiana* (78.63%)^31,34–36^. In contrast, lignin content was higher in our study compared with a previous analysis of *S. trifasciata* fibers that showed that Klason lignin made up 6% of the total dry mass^34^, about 42% lower than we found by py-MBMS. The differences in lignin composition may be due to differences in the type of lignin quantified in each technique (Klason lignin, acid-insoluble lignin; pyMBMS, total lignin) and sensitivity of the methods used^37^. For *Sansevieria* fibers, our report is the first to determine the chemical composition using high performance chromatography and mass spectroscopy. Differences in the chemical composition and morphology of the fibers in the two cultivars examined here seem to support the classification of very-soft fiber accession reported for *S. trifasciata* cv. Golden Hahnii^38^ and may account for differences in thermal and mechanical properties, which have so far been analyzed for Lorentii but not Hahnii^1,39,40^. In *S. cylindrica*, the fibers have slightly lower cellulose and hemicellulose contents and a 50% less lignin than in *S. ehrenbergii*, which causes notable differences in the density, tensile strength, Young’s modulus and elongation at break values of the fibers (reviewed by Lokantara et al.^41^). As lignin levels were similar in Lorentii and Hahnii analyzed here, potential differences in fiber tensile strength between cvs could be explained by differences in the cellulose and xylose contents found (Table 1). More research is necessary to support this hypothesis.

**Table 1:** Chemical composition of fibers in cvs Lorentii and Hahnii of *Sansevieria trifasciata*. Carbohydrates were quantified by HPAEC-PAD and lignin monomers by py-MBMS. Values are means ± SD. Means followed by different letters indicate a significant difference between fractions of both cultivars according to Tukey’s test (*p* ≤ 0.05).

Fraction	Mean ± SD (μg/mg of dry mass)	
	cv. Lorentii	cv. Hahnii
Glucose	418.030 ± 35.879	332.706 ± 45.463
Xylose	112.578 ± 16.384	87.360 ± 12.201
Rhamnose	0.931 ± 0.092	0.675 ± 0.082
Arabinose	2.405 ± 0.348	3.436 ± 0.228
Mannose	3.177 ± 1.381	3.324 ± 0.588
Cellobiose	2.460 ± 0.012	4.179 ± 0.486
Maltose	0.241 ± 0.070	0.327 ± 0.130
**Percent of total dry mass**		
S lignin	6.625 ± 0.403	5.725 ± 0.708
G lignin	5.275 ± 0.561	4.95 ± 1.078
S/G	1.267 ± 0.135	1.212 ± 0.381
C5:C6	0.98 ± 0.054	1.002 ± 0.099
Total lignin	14.8 ± 0.432	13.925 ± 0.525

### Lorentii and Hahnii differ in morphogenic responses to in vitro culture

**Figure 3:**
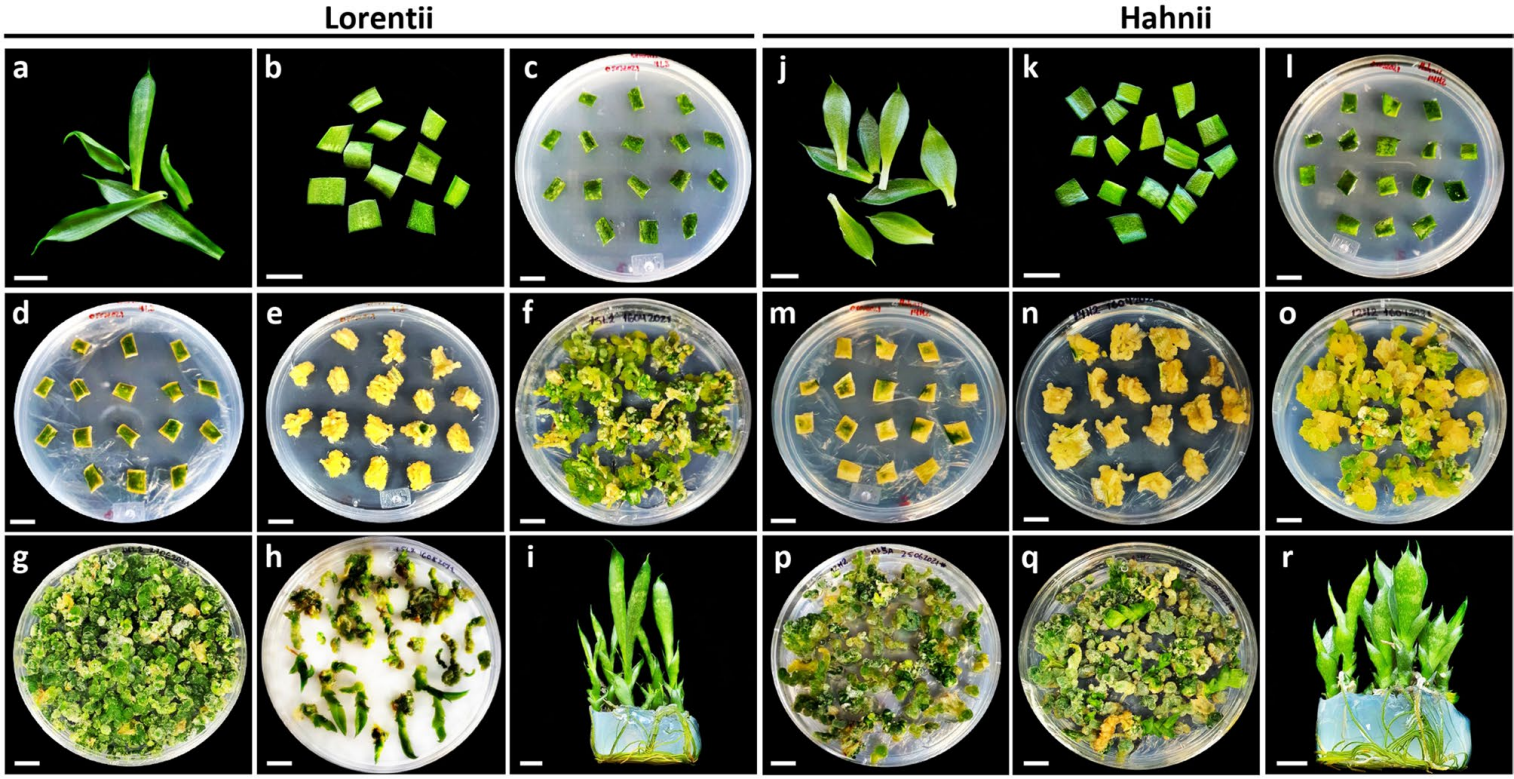
Overview of different steps for indirect de novo shoot production for cvs Lorentti and Hahnii of *Sansevieria trifasciata*. Establishment of (**a**, **b**, **c**, **j**, **k**, **l**) leaf-segment-derived culture, (**d**, **e**, **m**, **n**) primary calli, (**f**, **g**, **o**, **p**) organogenic calli, (**h**, **q**) shoot production and (**i**, **r**) shoot rooting.

**Figure 4:**
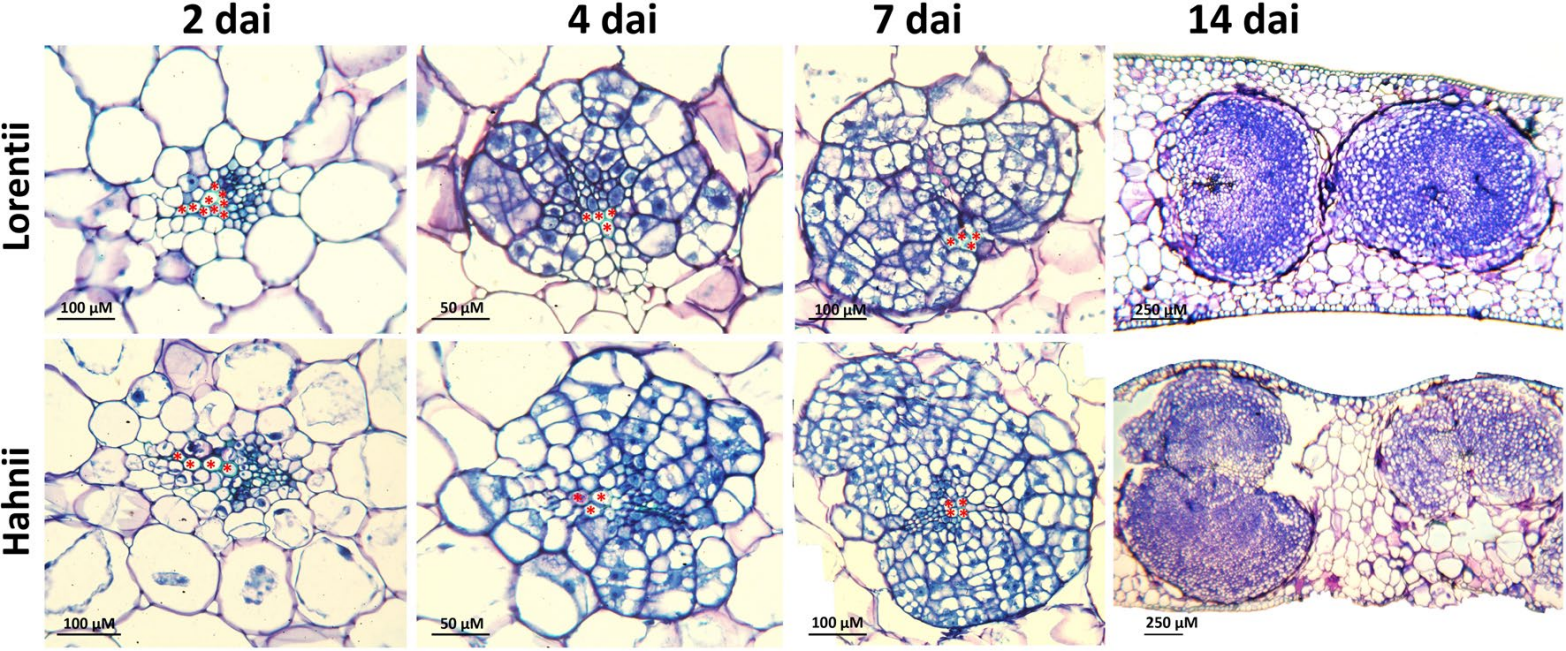
Time course of transdifferentiation events during in vitro callogenesis of *Sansevieria trifasciata*. Leaf explants cultured on 1X MS medium with 2 mg·Lˉ^1^ 2,4-D for callus induction were sampled, processed for microscopy, sectioned, and stained with a 1:1 1% methylene blue–1% azure B solution. Micrographs show high cell clusters surrounding ribbon fibers and vascular bundles. Asterisk = xylem cell. DAI = days after incubation.

To assess and compare the morphogenic response to in vitro culture of cvs Lorentii and Hahnii, we optimized an indirect organogenesis protocol for shoots (Supplementary Fig. 1), characterizing the initiation of organogenesis and the yields in each phase from callus induction to shoot production. An overview of these different phases is illustrated in Fig. 3. For both cultivars, primary callus was obtained from leaf segments after 42 days incubation (dai) in Murashige & Skoog^42^ (MS) medium supplemented with 2,4-D (Fig. 3a–e, j–n). The calli were friable and white-cream in color (Fig. 3e,n), typical of healthy, embryonic-like calli of monocots^43–46^. Although most chlorophyll content was lost and calli were already visible after 14 dai, 1 week earlier than previously reported for *S. trifasciata*^47^, a histological time-course analysis (2, 4, 7, and 14 dai) (Fig. 4) revealed that the transdifferentiation events in the ground tissue initiated very early under the in vitro culture conditions. At 4 dai, cross sections of initial leaf explants showed defined cell clusters, which were integrated with a high number of mitotic cells, scattered in the mesophyll tissue and exclusively surrounding vascular bundles and ribbon fiber caps (Fig. 4). These structures developed faster in cv Hahnii than in cv Lorentii. At 14 dai, the cell clusters had increased in volume and almost broke through the leaf epidermis (Fig. 4), explaining previous observations of calli located at the edges of *Sansevieria* leaf segments^47^. At the last time sampled, cell clusters also surrounded the structural fibers in both cultivars (Supplementary Fig. 2). In vitro cultured, leaf-derived calli can have different forms of ontogenesis. For example, in the woody monocot *Phoenix dactylifera,* calli are formed from fascicular parenchyma cells or perivascular sheath cells (PSCs) depending on the differentiation grade of the leaf explant used^48^. In *Coffea arabica*, a pro-embryogenic mass and callus originate from mitotically active cells found near vascular bundles^49^. Similarly in our study, *Sansevieria* leaf calli seem to derive exclusively from PSCs or totipotent cells surrounding fibers (sclerenchyma). PSCs may be considered pericycle-like cells, which are thought to serve as pluripotent stem cells for callus initiation and shoot organogenesis^50,51^, similarly to that observed in our results. In the case of the calli that formed around *Sansevieria* structural fibers, we presume they may be derived from remnant cells of the ground meristem, which is known to give rise to such extraxylary fibers^52^.

**Figure 5:**
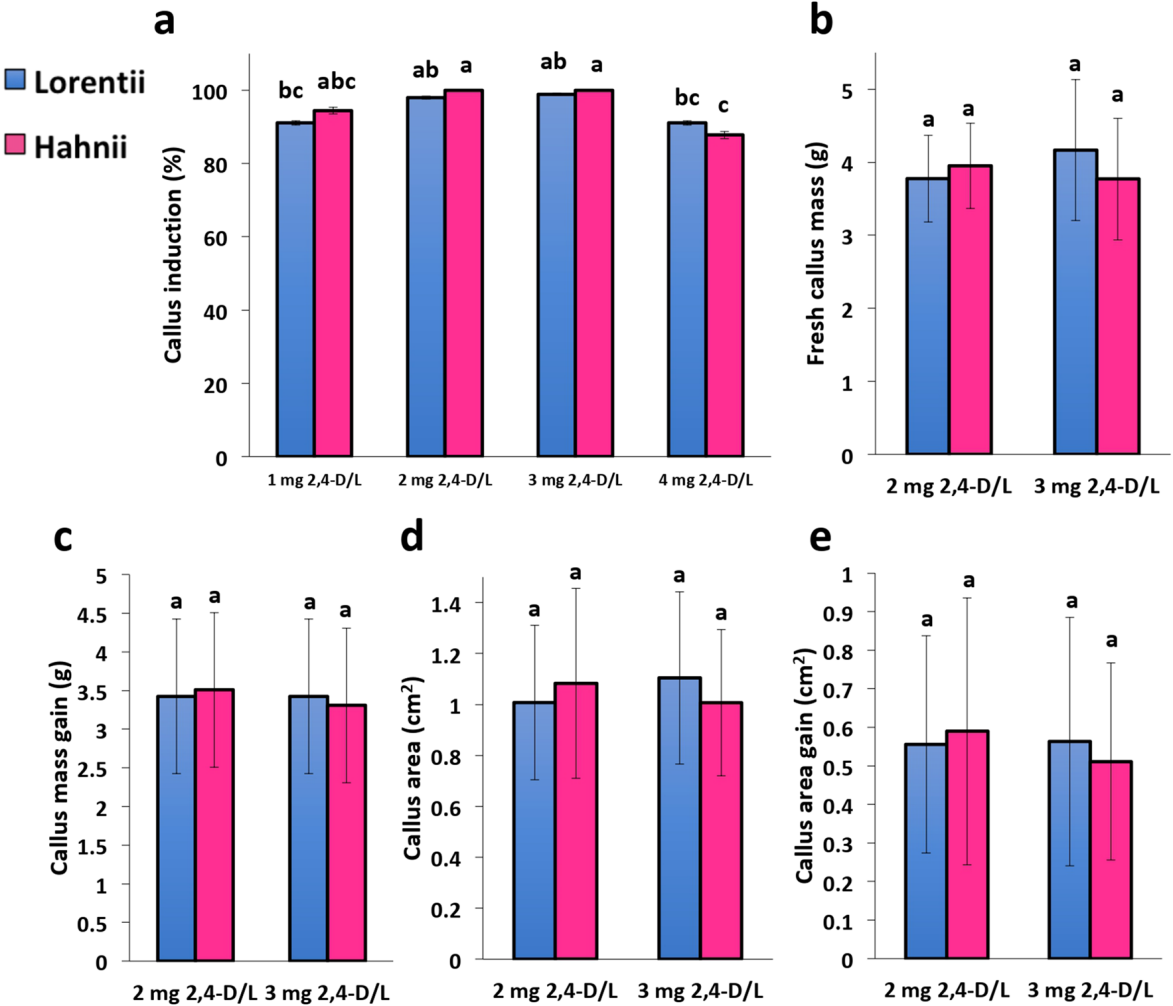
Effects of 2,4-D auxin on callus production for cvs Lorentii and Hahnii of *Sansevieria trifasciata*. (**a**) Callus induction, (**b**) fresh callus mass, (**c**) fresh mass gain index, (**d**) callus area, and (**e**) callus increase index were evaluated after 42 days of growth with different doses of 2,4-D. Values are means ± SD. Means with different letters are significantly different according to Tukey’s test (*p* ≤ 0.05).

Callogenesis performance of the two *Sansevieria* cultivars in response to four doses of 2,4-D (1, 2, 3, and 4 mg·L^-1^) was also assessed. By 42 days after treatment, callus induction for both cultivars showed a clear dose response (Fig. 5A). The doses most effective for inducing callus were 2 and 3 mg·L^-1^, but the highest dose reduced induction by 7.8% in Lorentii and 12.2% in Hahnii compared to the highest induction values obtained. In addition, Hahnii was more sensitive to 2,4-D (Fig. 5a). Our results for callogenesis induction are in line with previous reports for other monocot species such as agave, sugar cane, coconut, sorghum and desho; very high doses of 2,4-D considerably inhibited callus induction^45,53–56^.

The calli induced by the two most efficient doses were then weighed, calli areas calculated, and the gains obtained using the initial and final values for both parameters. After 6 weeks of treatment, in both cultivars, the callus mass and area under the two doses were similar in both snake varieties (Fig. 5b,d) (Table S1, S2). It is worth mentioning high gains in biomass and area during callogenesis of both Sansevieria cvs during short treatment time (Fig. 5c,e) (Table S1, S2). This high efficiency of callogenesis in snake plant is similar to that observed in *Campomanesia adamantium*^57^. In monocots, callogenesis performance has been quantified for only a few plant species so far. For example, in *Hyacinthus orientalis* up to 0.9 g callus/explant from leaf tissue was obtained after 6 weeks of culture on MS medium supplemented with 2 mg·L^-1^ of BAP and 1 mg·L^-1^ of kinetin^58^, whereas *Zingiber officinale* yielded up to 3.89 g of callus/g explant from leaf tissue after 30 days on MS medium with 2 mg·L^-1^ of 2,4-D and 1 mg·L^-1^ of BAP^59^. Although the callus induction conditions used for these two monocot species and those we evaluated here differ, the callogenesis performance obtained for *S. trifasciata* is outstanding; rapid cell transdifferentiation may conduct to high yields of callus biomass. This behavior of snake plants during callogenesis may be helpful for establishing cell cultures for biotechnological purposes using the method described here.

**Figure 6:**
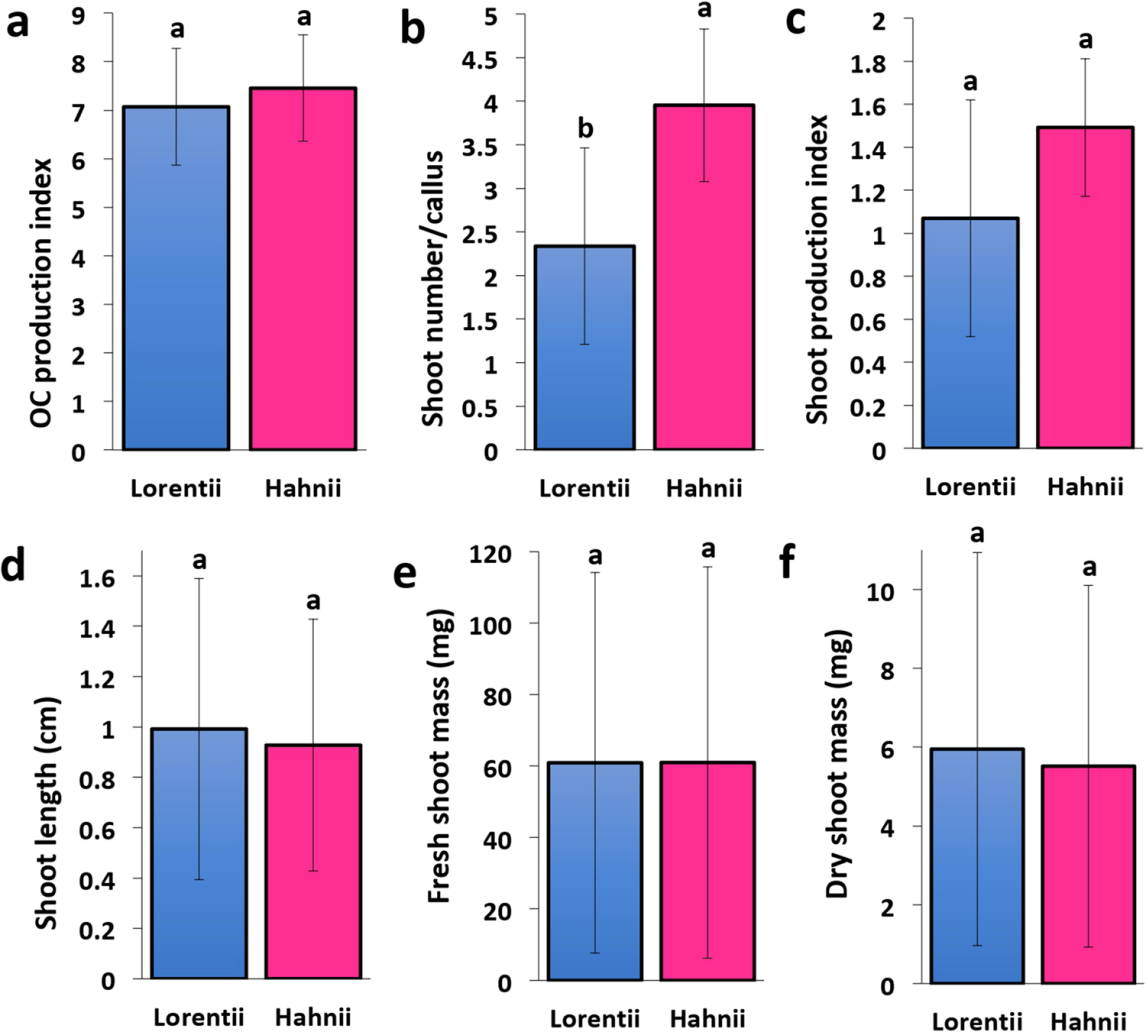
Shoot production by cvs Lorentii and Hahnii of *Sansevieria trifasciata*. (**a**) OC production index, (**b**) shoot number/callus, (**c**) shoot production index, (**d**) shoot length, (**e**) fresh shoot mass, and (**f**) dry shoot mass were evaluated after 4 weeks of growth on 1X MS medium + 6-benzylaminopurine (BAP) at 4.0 mg·Lˉ^1^. OC = Organogenic callus. Values are means ± SD. Means with different letters are significantly different according Tukey’s test (*p* ≤ 0.05).

For de novo shoot production, the primary calli were transferred to 1X MS medium supplemented with 4.0 mg·L^-1^ of BAP. After 4 weeks, primary calli produced chlorophyll and became organogenic. The indices of organogenic callus production for cvs Lorentii and Hahnii did not differ significantly (Fig. 6a) (Table S3). Shoots emerged after 8 weeks and were maintained in these conditions until week 12. Hahnii produced about 1.5 times more shoots than Lorentii (Fig. 6B) (Table S3), thus positively affecting the shoot production index calculated for both cultivars (Fig. 6c). When the length, fresh and dry mass of the shoots were compared between the two cultivars, no significant differences were found (Fig. 6d–f) (Table S3). Shoot production rates quantified here for both Lorentii and Hahnii were up to 4 times lower than reported for the same varieties previously^24^ and similar to that found by Hematharshini and Seran^50^, but faster than in both previous reports (4 weeks sooner). Our data suggest Hahnii is a cultivar with a stronger response to de novo shoot production than Lorentii, contradictory to the report of Yusnita et al.^24^.

**Figure 7:**
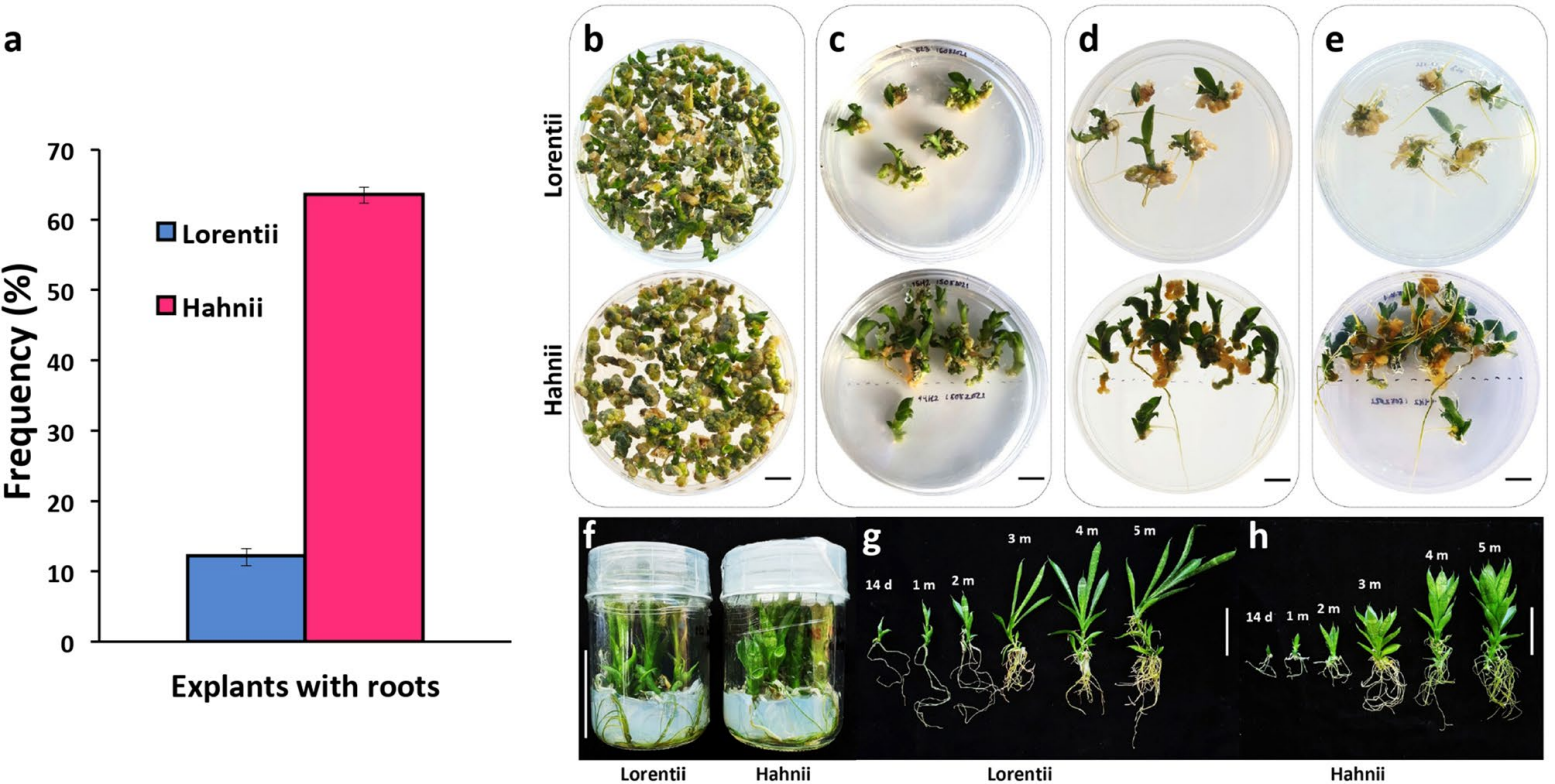
Rooting phase during micropropagation of cvs Lorentii and Hahnii of *Sansevieria trifasciata.* (**a**) Root formation in leaf explants of *Sansevieria trifasciata* incubated in basal 1X MS medium. Root development after (**b**) 12 weeks, (**c**) 16 weeks, and (**d**) (top view) and (**e**) (bottom view) 18 weeks on auxin-free 1X MS medium + 4.0 mg·Lˉ1 BAP. (**f**) Seedlings in hormone-free 1X MS medium. (**g**, **h**) Root development on plantlets growing on hormone-free 1X MS medium. Values are mean ± SD. Black bar = 1 cm, white bar = 5 cm.

Snake plants are also recognized for their rapid root production during conventional propagation using rhizomes or adult leaf cuttings^25,49,61^, suggesting that levels of endogenous auxins are sufficient for rhizogenesis. During the development of our protocol for in vitro multiplication, we observed that leaf explants on basal 1X MS medium yielded a higher frequency of root induction from Hahnii (63.3%) compared to Lorentii (12.2%) (Fig. 7a). Similarly, at the in vitro rooting stage, roots were generated when shoot cultures were grown with 4.0 mg·L^-1^ of BAP, without exogenous auxins, for both cultivars (Fig. 7b–e). Moreover, the root system was well-developed on plantlets grown in a hormone-free basal 1X MS medium (Fig. 7f–h). This last phase of our micropropagation protocol, an exogenous auxin-free rooting, contrast with those of Hematharshini and Seran^60^ and Simbolon^61^, who used 0.5 mg·Lˉ^1^ of NAA and 2.0 mg·L^-1^ of BAP to produce shoots and roots, respectively, and those of Kaur^25^, who used high doses of IBA (5.0–10.0 mg·L^-1^), and Sarmast et al.^22^ and Wahyuningsih et al.^62^, who immersed shoots for 3 s in an IBA solution (2000 mg·L^-1^), to induce rooting.

## Conclusions

To sum up, morphological and chemical characterization of fibers of two cultivars of S. trifasciata, Lorentii and Hahnii, was carried out. Histological analysis revealed a similar distribution pattern of structural and ribbon fibers in both cultivars but differences in their chemical composition. Lorentii showed higher cellulose and xylose contents than Hahnii, while the lignin levels were similar. These differences found may explain the different strength properties previously known for both cvs. Snake plants were also efficiently propagated using an indirect organogenesis protocol. The protocol proposed takes only 140 d from leaf explant to plantlets. Time-course histological analysis revealed early transdifferentiation events occurring around vascular bundles, which lead to a high organogenic callus production. Dosis-responses analysis showed that 2 and 3 mg·L-1 of BAP were the most efficient doses for callus induction, with performance levels similar in both cvs. When shoot production was characterized, Hahnii showed a stronger response to de novo shoot production than Lorentii. Our results showed S. trisfasciata can be easily and efficiently propagated via indirect organogenesis.

## Materials and methods

### Leaf morphometry and fiber chemical characterization

Cultivars Lorentii and Hahnii of *Sansevieria trifasciata* were grown in standard nursery conditions at Colegio de Postgraduados Campus Campeche (19°29′54.924″ N, − 90°32′43.656″ W). Culture and collect of plants for this study are in line with the national and institutional guidelines and legislation. Three adult plants of each cultivar were used as explant donors for in vitro culture. For morphometrics, adult plants (Lorentii, *n* = 11; Hahnii *n* = 30) were morphologically characterized by counting all leaves and measuring the length, width, area, fresh and dry mass of three fully expanded leaves.

For fiber characterization, adult leaves were autoclaved (121 °C, 15 PSI, 20 min), then the fibers were extracted manually using sharp tweezers, washed with tap water, and dried at 65 °C for 3 days. For monosaccharide composition fibers were processed as reported previously^48^. Fibers were freeze-dried overnight, ground and soaked in chloroform–methanol (2:1) for 1 h, then twice each in 70% ethanol for 1.5 h, 80% ethanol for 1 h, and 95% ethanol for 2 h at room temperature. Treated fibers were briefly washed with acetone and dried under a stream of air. For removing any residual starch during sampling, fibers were incubated with pancreatic α-amylase (16 U/g) (Sigma-Aldrich, Germany) overnight, and washed with acetone. Monosaccharides were obtained by Saeman hydrolysis; fibers were placed in 72% sulfuric acid for 3 h at room temperature, then the acid solution was diluted to 6% with deionized water and incubated at 100 °C for 3 h. Inositol was added to all samples as an internal standard before the acid hydrolysis. All hydrolysates were filtered through 0.2 µm nylon syringe filters, then analyzed using high performance anion exchange chromatography with pulsed amperometric detection (HPAEC-PAD) (ICS-3000, Dionex, Thermo Fisher Scientific, Sunnyvale, USA) equipped with a CarboPac PA1 column (4 × 250 mm, Dionex) as previously reported^50^.

For lignin composition (total lignin and monolignols), fiber samples were analyzed by pyrolysis-molecular beam mass spectrometry (py-MBMS) as reported previously^63,64^. Samples (1.5 to 3.0 mg) were prepared in duplicate; each was placed into a stainless metal cup, and single-shot pyrolyzed (Frontier Lab) at 500 °C to produce volatile compounds. The volatile compounds were analyzed for lignin using a molecular beam mass spectrometer (Extrel Core Mass Spectrometers). The raw data were processed through UnscramblerX 10.1 software to obtain the main components and raw lignin data. Also, NIST 8492 (lignin content, 26.2%) and Aspen standards were pyrolyzed and analyzed in the same manner and in the same batch as the unknown samples. Both standards were used for data quality control. Additionally, NIST 8492 was used to correct the raw lignin data.

### In vitro culture establishment

For in vitro culture, long strips of leaf segments were cut from the central midsection of fully expanded leaves. These leaf segments were hand-washed with running tap water and ordinary liquid soap for 10 min and then washed for 5 min with running water. In a laminar flow hood, they were then immersed for 10 min in 20% (v/v) commercial bleach (containing 5.25% sodium hypochlorite), followed by 1 min in 70% (v/v) ethanol. Next, they were soaked in an aqueous solution of cefotaxime 250 mg·L^-1^ and carbenicillin 50 mg·L^-1^ for 15 min. Finally, an axenic culture was established as follows: small leaf segments (0.5–1 cm^2^) were placed in Petri dishes that contained 25 mL of 1X MS medium (Caisson Labs) supplemented with 250 mg·L^-1^ cefatoxime, 3% (w/v) sucrose, 0.65% (w/v) agar (Micropropagation Agar Type I, Caisson Labs), and pH adjusted to 5.8 with KOH. The dishes were then incubated at 28 ± 2 °C with cool white LED lights (40 μmol m^-2^ s^-1^) and a 16 h light:8 h dark cycle.

### Callus induction

Small leaf segments (0.5–1 cm^2^) from plantlets cultured as previously reported^24^ were placed with the adaxial leaf side down on 1X MS agar prepared as described above but without antibiotics. For callus induction, 2,4-dichlorophenoxyacetic acid (2,4-D) (Caisson Labs) was tested at 0, 1, 2, 3, and 4 mg·L^-1^. For all treatments (completely randomized design, three replicates per treatment, 10 explants per replicate), plates were incubated as described above. Callus induction percentage was quantified after 6 weeks of culture. To determine the in vitro callogenesis performance for each cultivar of *S. trifasciata*, a second experiment (three replicates per treatment, 16 explants per replicate) was established using 2 and 3 mg·L^-1^ of 2,4-D doses, the best performing doses based on data from the callus induction experiment. Callus induction percentage, fresh callus mass and callus area were evaluated. For calculating callus area, calli were photographed and the calli areas were obtained using the free ImageJ software (NIH, US). Initial (IV) and final values (FV) of mass and area were then used to obtain gain values after treatment (Gain = FV-IV).

### De novo shoot organogenesis

For organogenic callus (OC) production, 6-week-old primary calli of both *Sansevieria* cultivars were transferred to 1X MS medium supplemented with BAP (4.0 mg·L^-1^) (Caisson Labs), 3% (w/v) sucrose, 0.65% (w/v) agar (Micropropagation type I, Caisson Labs), and pH adjusted to 5.8 with KOH. The BAP dosis used was selected according preliminary experiments for shoot proliferation in our laboratory and previous reports^65^. Incubation conditions were as described above. For each cultivar, five replicates were used per treatment with 16 explants per replicate in a completely randomized design. After 4 weeks, the capacity of OC production was determined by calculating the OC production index (fresh OC mass/fresh mass of primary callus). For de novo shoot formation, OC were subcultured for another 8 weeks in the same culture medium and incubated as described. To compare shoot production by each cultivar, we evaluated shoot number/callus, shoot production index (shoot number/fresh OC mass), shoot length, and fresh and dry shoot masses.

### Histological analysis

Transdifferentiation events during callus induction with 2 mg/L of 2,4-D, the best performing treatment according to the dose–response experiment described above, were examined microscopically in leaf samples collected at 2, 4, 7, and 14 days after the start of treatment. Samples were placed immediately in 4% paraformaldehyde (Sigma-Aldrich) (w/v) in 1X phosphate-buffered saline solution, and fixed at 4 °C for 1 week. The fixed tissues were then dehydrated in ethanolic series (30, 50, 70, 85, 96 and 100%) (2 h twice for each) at room temperature. Samples were embedded using an epoxy medium (JB-4, Polysciences) according to the manufacturer’s specifications. Resin-embedded samples were sectioned (8–10 µm thick) using an automatic rotatory microtome (Leica RM2255). Cross sections were stained using a 1:1 aqueous solution of 1% w/v methylene blue and 1% w/v azure B^66^ for 20 s at room temperature and washed with distilled water, dried at 50ºC overnight and mounted in synthetic resin (Hycel, 7987). Leica DM2000 and Carl Zeiss Axioakop 2 Plus microscopes were used for imaging the stained sections. Lignin and cellulose were analyzed by confocal laser scanning microscopy (Leica TCS SP8). Lignin was detected by autofluorescence (405 nm excitation, 414–501 nm emission) and cellulose was detected by staining with an aqueous solution of propidium iodide (10 mg mL^-1^) (Sigma-Aldrich, Germany) (488 nm excitation, 642–748 nm emission).

### Statistical analyses and image processing

All data, reported as means ± standard deviations (SD), were analyzed as a completely randomized factorial design. One-way independent ANOVA analyses were carried out using SAS software v. 9.2 (SAS Institute, Cary, NC, USA). For comparisons between treatments, Tukey’s honestly significant difference (HSD) tests were performed (*p* ≤ 0.05). Figures were prepared using Remove.bg (Kaleido Al GmbH, Vienna, Austria), PowerPoint (Microsoft, Redmond, USA) and iLovePDF (PDF-Tools, Barcelona, Spain).

## Supplementary Information

Supplementary Information 1.

Supplementary Information 2.

Supplementary Information 3.

Supplementary Information 4.

## Data Availability

All data generated or analyzed during this study are included in this published article [and its supplementary information files].
