# Supplementary material for: Indirect organogenesis for high frequency shoot regeneration of two cultivars of Sansevieria trifasciata Prain differing in fiber production
Source: Sci Rep. 2022 May 20;12:8507. doi: 10.1038/s41598-022-12640-4 (PMC9122912; doi:10.1038/s41598-022-12640-4)
Supplement: Supplementary file 1 — Supplementary Information 1. [file 41598_2022_12640_MOESM1_ESM.docx]

**Indirect organogenesis to generate a high frequency of shoots in two cultivars of *Sanseviera trifasciata* Prain that differ in fiber production**

Eleazar García-Hernández, Maribel M. Loera-Quezada, Dalia C. Morán-Velázquez, Mercedes G. López, Manuel A. Chable-Vega, Alberto Santillán-Fernández, Hilda A. Zavaleta-Mancera, John Z. Tang, Parastoo Azadi, Enrique Ibarra-Laclette & Fulgencio Alatorre-Cobos

**
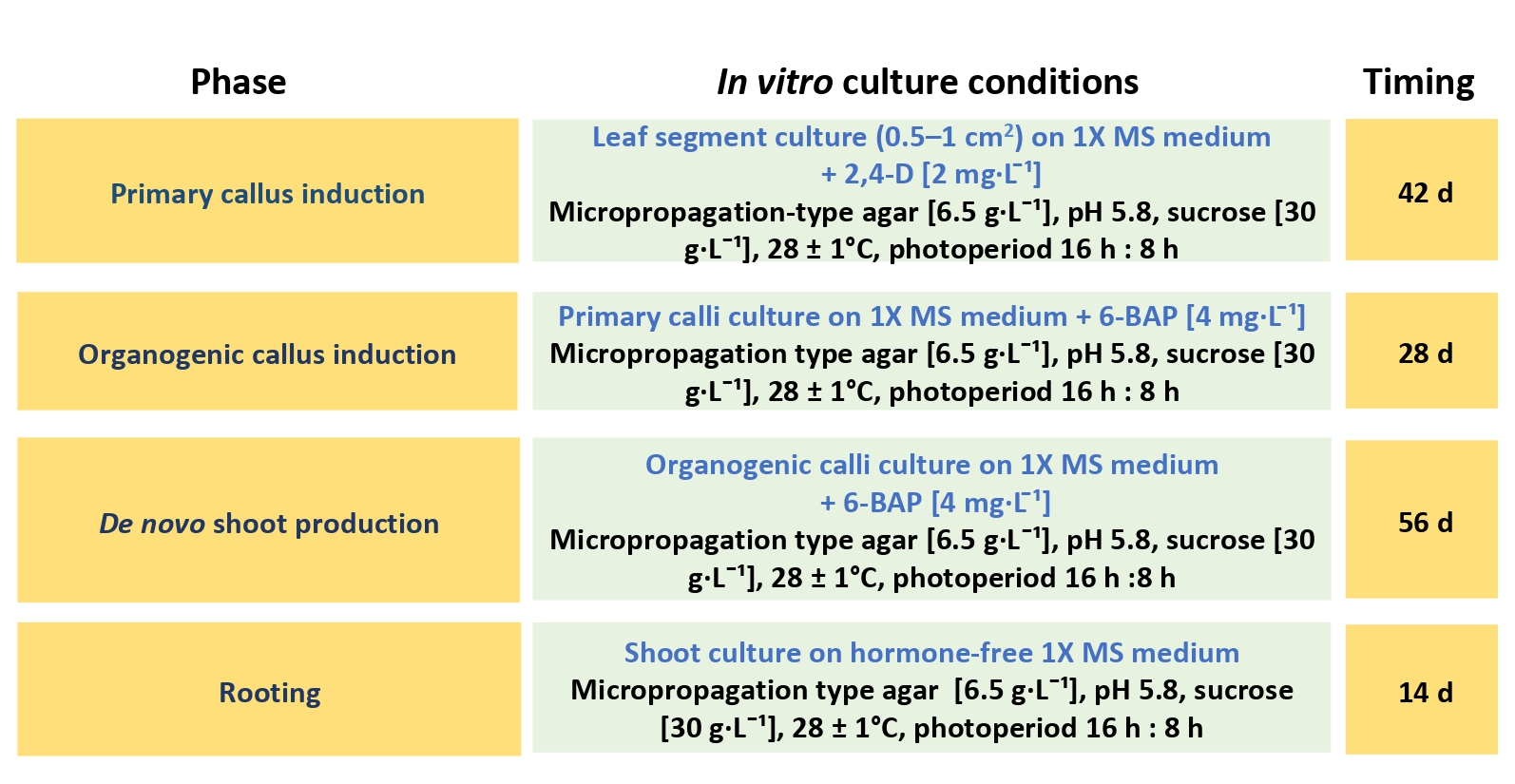
**

**Supplementary Figure 1**. Protocol for callogenesis and *de novo* shoot production for cvs Lorentii and Hahnii of *Sansevieria trifasciata*.

**
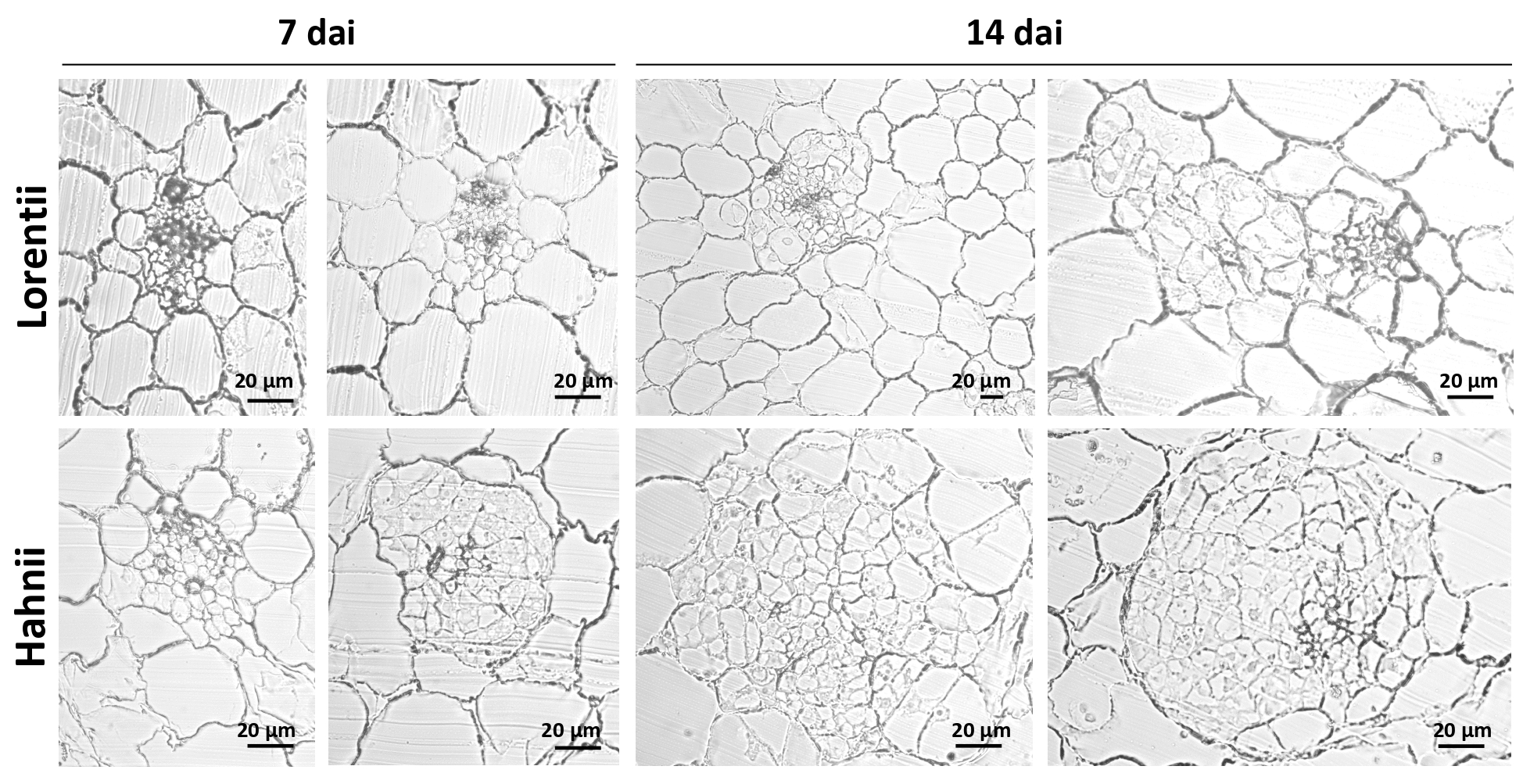
**

**Supplementary Figure 2**. Development of cell clusters surrounding leaf structural fibers by cvs Lorentii and Hahnii of *Sansevieria trifasciata* during callus induction on 1X MS medium + 2 mg L^-1^ of 2,4-D. dai = days after induction.
