# Supplementary material for: Indirect organogenesis for high frequency shoot regeneration of two cultivars of Sansevieria trifasciata Prain differing in fiber production
Source: Sci Rep. 2022 May 20;12:8507. doi: 10.1038/s41598-022-12640-4 (PMC9122912; doi:10.1038/s41598-022-12640-4)
Supplement: Supplementary file 2 — Supplementary Information 2. [file 41598_2022_12640_MOESM2_ESM.docx]

**Supplementary Table 1.** Data of fresh callus mass, callus mass gain, callus area, and callus area gain of two cultivars of *Sansevieria trifasciata* under two doses of 2,4-D doses (Data presented here were used for Fig. 5b-e)

| Cultivar/Treatment | LS fresh mass (g) | Callus fresh mass (g) | Callus mass gain (g) | LS initial area (cm^2^) | Callus area (cm^2^) | Area gain (cm^2^) |
| --- | --- | --- | --- | --- | --- | --- |
| Lorentii 2 mg·Lˉ¹ | 0.351 ± 0.055 | 3.775 ± 0.593 | 3.423 ± 0.610 | 0.450 ± 0.108 | 1.007 ± 0.303 | 0.555 ± 0.281 |
| Lorentii 3 mg·Lˉ¹ | 0.390 ± 0.060 | 4.16 ± 0.963 | 3.749 ± 0.934 | 0.540 ± 0.129 | 1.104 ± 0.338 | 0.563 ± 0.322 |
| Hahnii 2 mg·Lˉ¹ | 0.442 ± 0.071 | 3.949 ± 0.584 | 3.508 ± 0.566 | 0.493 ± 0.115 | 1.082 ± 0.372 | 0.589 ± 0.346 |
| Hahnii 3 mg·Lˉ¹ | 0.459 ± 0.052 | 3.768 ± 0.835 | 3.307 ± 0.830 | 0.495 ± 0.109 | 1.006 ± 0.286 | 0.511 ± 0.256 |

Leaf segment = LS
