# Supplementary material for: Indirect organogenesis for high frequency shoot regeneration of two cultivars of Sansevieria trifasciata Prain differing in fiber production
Source: Sci Rep. 2022 May 20;12:8507. doi: 10.1038/s41598-022-12640-4 (PMC9122912; doi:10.1038/s41598-022-12640-4)
Supplement: Supplementary file 3 — Supplementary Information 3. [file 41598_2022_12640_MOESM3_ESM.docx]

**Supplementary Table 2.** ANOVA and Tukey’s HSD *t* results of data of callus induction percentage, fresh callus mass, callus mass callus gain, callus area, and callus area gain (Data presented here were also used in Fig. 5)

| Callus induction percentage under different doses of 2,4-D doses (Fig. 5a) | | | | | | | | | | |
| --- | --- | --- | --- | --- | --- | --- | --- | --- | --- | --- |
| Source | SS | Df | MS | *F*-test | *P* | Test: Tukey α = 0.05 LSD = 8.0457, Error: 27.5 df: 80 | | | | |
| Model | 131738.8889 | 9 | 14637.6543 | 532.28 | <0.0001 | Induction percentage | Mean |  | SE | Grouping |
| Error | 2200.0000 | 80 | 27.5000 |  |  | Hahnii 3 mg·L^-1^ | 100.0000 |  | 0 | a |
| Total | 133938.8889 | 89 |  |  |  | Hahnii 2 mg·L^-1^ | 100.0000 |  | 0 | a |
|  |  |  |  |  |  | Lorentii 3 mg·L^-1^  Lorentii 2 mg·L^-1^  Hahnii 1 mg·L^-1^  Lorentii 1 mg·L^-1^  Lorentii 4 mg·L^-1^  Hahnii 4 mg·L^-1^ | 98.889  97.778  94.444  91.111  91.111  87.778 |  | 0.333  0.440  0.881  0.600  0.600  0.971 | ab  ab  abc  bc  bc  c |
| Fresh callus mass under two doses of 2,4-D doses (Fig. 5b) | | | | | | | | | | |
| Source | SS | Df | MS | *F*-test | *P* | Test: Tukey α = 0.05 LSD = 0.6781, Error: 0.54915 df: 64 | | | | |
| Model | 1.56356616 | 3 | 0.52118872 | 0.95 | 0.4223 | Fresh callus mass | Mean |  | SE | Grouping |
| Error | 35.1459399 | 64 | 0.54915553 |  |  | Lorentii 3 mg·L^-1^ | 4.1651 |  | 0.9636037 | a |
| Total | 36.70952015 | 67 |  |  |  | Hahnii 2 mg·L^-1^ | 3.9495 |  | 0.58475861 | a |
|  |  |  |  |  |  | Lorentii 2 mg·L^-1^  Hahnii 3 mg·L^-1^ | 3.7752  3.7683 |  | 0.593368  0.835005892 | a  a |
| Calculated callus gain under two doses of 2,4-D doses (Fig. 5c) | | | | | | | | | | |
| Source | SS | Df | MS | *F*-test | *P* | Test: Tukey α = 0.05 LSD = 0.6692, Error: 0.53477 df: 64 | | | | |
| Model | 1.55566191 | 3 | 0.51855397 | 0.97 | 0.4126 | Weight callus gain | Mean |  | SE | Grouping |
| Error | 34.22557406 | 64 | 0.53477459 |  |  | Lorentii 3 mg·L^-1^ | 3.7497 |  | 0.934212169 | a |
| Total | 35.78123597 | 67 |  |  |  | Hahnii 2 mg·L^-1^ | 3.5086 |  | 0.566581151 | a |
|  |  |  |  |  |  | Lorentii 2 mg·L^-1^ | 3.4238 |  | 0.610088045 | a |
|  |  |  |  |  |  | Hahnii 3 mg·L^-1^ | 3.3075 |  | 0.830987134 | a |
| Callus area under two doses of 2,4-D doses (Fig. 5d) | | | | | | | | | | |
| Source | SS | Df | MS | *F*-test | *P* | Test: Tukey α = 0.05 LSD = 0.1003, Error: 0.108532 df: 574 | | | | |
| Model | 1.09774304 | 3 | 0.36591435 | 3.37 | 0.0183 | Callus area | Mean |  | SE | Grouping |
| Error | 62.29746757 | 574 | 0.10853217 |  |  | Lorentii 3 mg·L^-1^ | 1.10428 |  | 0.338244412 | a |
| Total | 63.39521060 | 577 |  |  |  | Hahnii 2 mg·L^-1^ | 1.08279 |  | 0.372995391 | a |
|  |  |  |  |  |  | Lorentii 2 mg·L^-1^ | 1.00747 |  | 0.303624832 | a |
|  |  |  |  |  |  | Hahnii 3 mg·L^-1^ | 1.00674 |  | 0.286795576 | a |
| Callus area gain under two doses of 2,4-D doses (Fig. 5e) | | | | | | | | | | |
| Source | SS | df | MS | *F*-test | *P* | Test: Tukey α = 0.05 LSD = 0.0932, Error: 0.09368933 df: 574 | | | | |
| Model | 0.42975691 | 3 | 0.14325230 | 1.53 | 0.2059 | Area gain | Mean |  | SE | Grouping |
| Error | 53.77767751 | 574 | 0.09368933 |  |  | Hahnii 2 mg·L^-1^ | 0.58959 |  | 0.34611731 | a |
| Total | 54.20743442 | 577 |  |  |  | Lorentii 3 mg·L^-1^ | 0.56331 |  | 0.322439701 | a |
|  |  |  |  |  |  | Lorentii 2 mg·L^-1^ | 0.55573 |  | 0.281639053 | a |
|  |  |  |  |  |  | Hahnii 3 mg·L^-1^ | 0.51146 |  | 0.256153524 | a |
