# Supplementary material for: Indirect organogenesis for high frequency shoot regeneration of two cultivars of Sansevieria trifasciata Prain differing in fiber production
Source: Sci Rep. 2022 May 20;12:8507. doi: 10.1038/s41598-022-12640-4 (PMC9122912; doi:10.1038/s41598-022-12640-4)
Supplement: Supplementary file 4 — Supplementary Information 4. [file 41598_2022_12640_MOESM4_ESM.docx]

**Supplementary table 3.** ANOVA and Tukey’s HSD *t* results for data on organogenic callus production index, shoot number/callus, shoot production index, shoot length, fresh and dry shoot mass of two cultivars of *Sansevieria trifasciata* (Data presented here were used in Fig. 6)

| OC production index (Fig. 6a) | | | | | | | | | | |
| --- | --- | --- | --- | --- | --- | --- | --- | --- | --- | --- |
| Source | SS | Df | MS | *F*-test | *P* | Test: Tukey α = 0.05 LSD = 1.4799, Error: 1.32348032 df: 10 | | | | |
| Model | 0.44482568 | 1 | 0.44482568 | 0.34 | 0.5749 | OC production | Mean |  | SE | Grouping |
| Error | 13.23480316 | 10 | 1.32348032 |  |  | Hahnii | 7.4530 |  | 1.094436382 | a |
| Total | 13.67962884 | 11 |  |  |  | Lorentii | 7.0680 |  | 1.20381462 | a |
|  |  |  |  |  |  |  |  |  |  |  |
| Shoot number/callus (Fig. 6b) | | | | | | | | | | |
| Source | SS | Df | MS | *F*-test | *P* | Test: Tukey α = 0.05 LSD = 1.2989, Error: 1.01948495 df: 10 | | | | |
| Model | 7.85431134 | 1 | 7.85431134 | 7.70 | 0.0196 | Shoot number/callus | Mean |  | SE | Grouping |
| Error | 10.19484954 | 10 | 1.01948495 |  |  | Hahnii | 3.954166667 |  | 0.876665082 | a |
| Total | 18.04916088 | 11 |  |  |  | Lorentii | 2.336111111 |  | 1.127132752 | b |
| Shoot production index (Fig. 6c) | | | | | | | | | | |
| Source | SS | Df | MS | *F*-test | *P* | Test: Tukey α = 0.05 LSD = 0.5789, Error: 0.20252003 df: 10 | | | | |
| Model | 0.53578248 | 1 | 0.53578248 | 2.65 | 0.1349 | Shoot production index | Mean |  | SE | Grouping |
| Error | 2.02520033 | 10 | 0.20252003 |  |  | Hahnii | 1.49217889 |  | 0.32012649 | a |
| Total | 2.56098281 | 11 |  |  |  | Lorentii | 1.069574867 |  | 0.550053722 | a |
| Shoot length (Fig. 6d) | | | | | | | | | | |
| Source | SS | Df | MS | *F*-test | *P* | Test: Tukey α = 0.05 LSD = 0.1017, Error: 0.2916256 df: 455 | | | | |
| Model | 0.4499457 | 1 | 0.4499457 | 1.54 | 0.2148 | Shoot length | Mean |  | SE | Grouping |
| Error | 132.6896604 | 455 | 0.2916256 |  |  | Lorentii | 0.991620112 |  | 0.598018405 | a |
| Total | 133.1396061 | 456 |  |  |  | Hahnii | 0.927338129 |  | 0.499213208 | a |
| Shoot fresh mass (Fig. 6e) | | | | | | | | | | |
| Source | SS | df | MS | *F*-test | *P* | Test: Tukey α = 0.05 LSD = 0.0009, Error: 0.00002254 df: 455 | | | | |
| Model | 0.00002044 | 1 | 0.00002044 | 0.91 | 0.3415 | Fresh weight | Mean |  | SE | Grouping |
| Error | 0.01025727 | 455 | 0.00002254 |  |  | Lorentii | 0.005949832 |  | 0.00498451 | a |
| Total | 0.01027772 | 456 |  |  |  | Hahnii | 0.005516547 |  | 0.004589583 | a |
| Shoot dry mass (Fig. 6f) | | | | | | | | | | |
| Source | SS | df | MS | *F*-test | *P* | Test: Tukey α = 0.05 LSD = 0.0102, Error: 0.00293617 df: 455 | | | | |
| Model | 0.00000052 | 1 | 0.00000052 | 0.00 | 0.9894 | Dry weight | Mean |  | SE | Grouping |
| Error | 1.33595711 | 455 | 0.00293617 |  |  | Hahnii | 0.060994964 |  | 0.054772582 | a |
| Total | 1.33595764 | 456 |  |  |  | Lorentii | 0.060925698 |  | 0.05326145 | a |

OC= Organogenic callus
